# A Disquieting Document

**Published:** 1921-02-12

**Authors:** 


THE PUBLIC WELL-BEING—(*continued*).

### A Disquieting Document.

The bulky and exhaustive report on the Pandemic of Influenza, just issued from H.M. Stationery Office by the Ministry of Health, makes available some valuable information for those engaged in medical research, but in some respects it is a rather disquieting document. It is the first intensive study of the great influenza scourge of 1918-1919 which swept across both Asia and Europe and claimed in the space of a few months a larger number of victims than fell during the whole of the European war.

In England and Wales alone the death toll reached 151,466. The authorities, referring to our British experience, state that many of the victims of the disease were young men, and the subsequent loss of the productive power of the community constitutes a grave social problem. But, apart altogether from the actual deaths, the visitation left in its wake a long trail of sickness. There are numberless men and women whose constitutions have been undermined and whose vitality has been sapped by the after-effects of this dreaded plague. The loss thus entailed to the health of the nation is one not to be computed in terms of recorded statistics. "The conclusion to which we are led," continue the authors of the report, "is that the generation of a great pestilence such as influenza or pneumonic plague is dependent upon disturbance of social order, involving for absolutely large numbers of human beings the endurance of conditions of insalubrity which afford the invading parasites a suitable field of modification. One thing appears to be reasonably certain, and that is that until a universal improvement in the standard of comfort and the condition of life is secured there will be no prospect of effectively mitigating the incidence of this deadly complaint."

It is only too obvious from this report that until the baffling mystery of influenza is solved all the national health services of the world will periodically have to face a problem full of the gravest anxiety. It is well that the conclusion of the official researches should be generally known and understood, for it will only be by the co-operation of the public with the authorities that recurring epidemics can be confronted with all the resources available.

